# Oral diseases and oral health related behaviors in adolescents living in Maasai population areas of Tanzania: a cross-sectional study

**DOI:** 10.1186/s12887-019-1655-8

**Published:** 2019-08-07

**Authors:** Lutango D. Simangwa, Anne N. Åstrøm, Anders Johansson, Irene K. Minja, Ann-Katrin Johansson

**Affiliations:** 1 Department of Clinical Dentistry - Cariology, Faculty of Medicine, University of Bergen, Bergen, Norway; 2 Department of Clinical Dentistry – Community Dentistry, Faculty of Medicine, University of Bergen, Bergen, Norway; 3 Department of Clinical Dentistry - Prosthodontics, Faculty of Medicine, University of Bergen, Bergen, Norway; 4 Department of Restorative Dentistry, School of Dentistry, Muhimbili University of Health and Allied Sciences, Dar Es Salaam, Tanzania

**Keywords:** Adolescents, Maasai populated areas, Oral diseases, Oral health related behaviors

## Abstract

**Background:**

Oral diseases, such as dental caries, tooth wear, dental erosion and periodontal diseases are major health problems in many societies. The study aim was to explore the association between oral health related behaviors and the presence of oral diseases in adolescents living in Maasai population areas in the northern part of Tanzania.

**Methods:**

A cross sectional study was conducted in 2016 using one stage cluster sample design. A total of 989 adolescents were invited and 906 (91.6%; (Maasais *n* = 721, non Maasais *n* = 185) accepted the invitation and completed an interview and clinical oral examination in a school setting (mean age 13.4 years, SD 1.2, range 12–17 years). Chi-square test, bivariate analysis and logistic regression were performed to analyze data.

**Results:**

Logistic regression revealed that: adolescents with low frequency of tooth cleaning (OR = 10.0, CI 4.3–20.0) was associated with poor oral hygiene and that more regular tooth cleaning (OR = 0.1, CI 0.04–0.14) and the use of plastic type of tooth brush (OR = 0.7, CI 0.53–0.99) were associated with less gingival bleeding. High consumption of biscuits (OR = 2.5, CI 1.7–3.8) was associated with presence of dental caries and the use of magadi (OR = 24.2, CI 11.6–50.6) as a food additive was the covariate for more severe dental fluorosis (TF grade 5–9). Regular intake of carbonated soft drinks (OR = 1.6, CI 1.1–2.5) and regular tooth cleaning (OR = 1.7, CI 1.1–2.6) were independently associated with dental erosion. Using teeth as a tool for: biting nails (OR = 1.9, CI 1.4–2.4), opening soda (OR = 1.8, CI 1.4–2.4) and holding needles (OR = 1.6, CI 1.3–2.1) were covariates for tooth wear. Adolescents who reported to clench/grind their teeth (OR = 2.3, CI 1.5–3.7) was the only covariate for TMD. In several of the investigated factors, there were significant differences between the Maasai and non Maasai ethnic groups.

**Conclusion:**

Oral health related behaviors have a significant impact on oral diseases/conditions among adolescents attending primary schools in Maasai population areas with obvious differences in behavior between the Maasai and non Maasai ethnic groups. There is a need for addressing oral health and to encourage behaviors that promote good oral health and dental care service utilization in this society.

## Background

Oral diseases are among the major public health problems in many societies [1, 2]. Research evidence suggest that oral health related behaviors, for example dietary habits and oral hygiene practices, are strongly related to the occurrence of oral diseases [3, 4]. Thus, oral diseases can often be avoided by modification of certain behaviors, for instance, the consumption of sugary or acidic foods and drinks, the exposure to fluoride and the level of oral hygiene practices [3–5]. Regarding the extent and severity of oral diseases, studies among adolescents aged 10–14 years from Tanzania (2010), Kenya (2012) and Uganda (2003) have revealed low prevalence of dental caries and the mean DMFT ranging from 0.3 to 0.7 [6–8]. Dental fluorosis is quite common in some regions of East African countries with a prevalence of 100% [9]. The prevalence of more severe dental fluorosis (grade ≥ V) according to Thylstrup Fejerskov Index in adolescents 10–15 year olds in Kenya (2009) and Tanzania (2000) was found to be 48% and 10–34%, respectively [10, 11]. Severe dental erosion in adolescents have been reported worldwide with prevalence varying from 3 to 20% [12, 13]. There is no information on dental erosion from sub-Saharan Africa in adolescents. Temporomandibular disorders (TMD) are a significant public health problems reported to affect 3–11% of the population [14]. In sub-Saharan Africa, studies on TMD are rare, studies from Tanzania and Nigeria reported that 67% of the Tanzanian adults and 26% of the Nigerian young adults had some signs and symptoms of TMD [15, 16].

In addition to individual oral health related behaviors, access to professional oral health care and lack of knowledge in combination with shortage of economic resources are influencing the possibility for the individuals to maintain an adequate oral health [17, 18]. It has to be considered that treatment of oral diseases is expensive, and in many developing countries reflected by the fact that the costs of treating children’s dental caries alone would exceed the total health care budget for those children [19]. The majority of the population in sub-Saharan Africa do not have access to appropriate oral health care services, especially those living in the rural areas [20, 21]. As a consequence, untreated oral diseases might lead to pain, problems with eating/chewing, smiling as well as speaking and limiting individual’s daily activities and quality of life [2]. In addition, other difficulties are related to life experiences and psychosocial factors including age, gender, education, ethnicity, language, anxiety, feeling of vulnerability, treatment need and cost, disability, beliefs and attitude towards oral health [20, 22–24].

In Tanzania there are more than 125 different ethnic groups. The Maasais is one of them living around the Arusha region in the Northern part of Tanzania. Historically the Maasai are believed to have originated from Sudan and to have migrated through the river Nile into Kenya and then further on in- to Tanzania. Their migration is due to their traditionally nomadic lifestyle whereby they move from one region to another searching for greener pastures for their livestock. Due to this kind of lifestyle they do not have permanent houses [25]. Historically, the Maasais original survival depends much on pastoralism, but today their way of living is increasingly moving towards agro-pastoralism [26]. This follows that their traditional diet, consisting of mainly meat and milk products, nowadays more often will include farm products, such as maize, rice and potatoes. Changes in dietary patterns, may expose the Maasai society to a different pattern of both oral and general diseases [2]. Previous report from Tanzania have shown that oral diseases associate with oral health related behaviors [27]. One previous study have shown that oral diseases among young adolescents in the Maasai population areas in Tanzania are common [28]. However, associations between oral diseases/ and oral health related behaviors have not recently been investigated in this particular socio-cultural context.

This study aims to explore associations between oral health related behaviors and the presence of oral diseases, adjusted for socio-demographic factors, focusing on adolescents living in Maasai population areas in the northern part of Tanzania.

## Methods

### Sample size

The sample size 845 was estimated based on the assumption that the prevalence of dental erosion, among the adolescents would be 50%, a margin error of 5% and confidence intervals of 95%.

### Sampling technique

A cross-sectional study was carried out, from June to November 2016, among adolescents living in Maasai population areas of Monduli and Longido districts, Arusha region in the Northern part of Tanzania. A list of all primary schools comprising public (urban and rural) and private schools (total of 100 schools) from both districts was obtained from the district education department. From this list all urban and private primary schools were excluded, leaving 66 eligible rural public primary schools (38 schools from Monduli and 28 from Longido district) for the sample frame. Urban and private schools were excluded because our main aim was to capture a maximum number of adolescents from pastoral societies living in remote rural areas and the majority of them cannot afford to attend private schools as they are very expensive. Out of these 66 schools, 23 schools were randomly selected (13 from Monduli and 10 from Longido) using a one-stage cluster sample design with schools as the primary sampling unit and random number generator software. From each randomly selected school a class expected to contain adolescents aged 12–14 years was identified (grade 6). All pupils in the selected classes meeting the inclusion criteria for age were invited to participate in the study. Further details of the sampling procedure has been described elsewhere [28].

### Interview

In this paper, some of the methods used have been described in our previous work [28]. Trained medical nurses, native of the study area, who spoke both Swahili and Maasai language fluently, performed a face- to- face interview with each participant at school outside or inside the classroom, depending on availability. The participants were interviewed using a questionnaire with closed- and open-ended questions. The questions were constructed in English, translated into Swahili and back-translated to English independently by qualified translators from the University of Dar Es Salaam, Tanzania. A pilot study (*n* = 50, age 12–14), testing the interview schedule, took place before the actual fieldwork regarding wording, meaning, and content on each item, and appropriateness of format. Participants of this pre-testing were not included in the main study [28].

Socio-demographic factors were assessed in terms of district of residence, age, sex, ethnicity, wealth index and mother’s education. Details of the assessment of sociodemographic factors have been described elsewhere [28]. Wealth index was assessed by asking the presence/absence of durable household assets indicative of family wealth (i.e. radio, television, refrigerator, mobile telephone, cupboard, bicycle and motorcycle) was recorded as (Yes) “available and in working condition” or (No) “not available and/or not in working condition.” Oral health related behaviors were assessed in terms of dietary habits, oral hygiene practices, alcohol, tobacco use, dental service utilization, sources of fluoride ingestion and use of teeth in their daily activities. Dietary habits were assessed by asking *“How often do you eat” a particular type of food* e.g. *sweets, biscuits, sugar, honey, maize stiff porridge, rice, cassava, sweet potatoes, Irish potatoes, meat, boiled blood, beans, fish, groundnuts* and *“How often do you drink” a particular type of drink* e.g. *carbonated soft drink, fruit drink, water plain, water with sugar, tea plain, tea with sugar, blood from animals, fresh milk from animals and soured milk?* Oral hygiene practice was assessed by asking two questions: *“How often do you clean your teeth”* and *“Do you use toothpaste during tooth cleaning”?* For both the dietary habits and oral hygiene practices the response categories were 0 = never, 1 = once to several times per month, 2 = once weekly, 3 = two or more times per week and 4 = daily. During analysis, the categories were dichotomized into 0 = low frequency/at most once per week (with options 0, 1 and 2) and 1 = high frequency/at least twice per week (with options 3 and 4). The type of cleaning instrument used was assessed by asking *“What type of cleaning instrument do you most often use to clean your teeth”?* The responses were *plastic type, chewing stick, charcoal or don’t have*. In logistic regression the options of charcoal and don’t have were omitted due to fewer cases. The tobacco and alcohol habits were assessed by asking *“How often do you smoke/chew tobacco”* and *“How often do you drink alcohol”?* The response categories were 0 = never, 1 = once to several times per month, 2 = once weekly, 3 = two or more times per week and 4 = daily. During analysis, the categories were dichotomized into 0 = never (with option 0) and 1 = irregularly (with options 1, 2, 3 and 4). Dental services utilization was assessed by asking *“Before today, have you ever visited a dentist/dental therapist for toothache”* and *“Before today, have you ever visited a dentist/dental therapist for checkup”?* The responses were *“yes”* or *“no”*. If “*yes*”, then the type of treatment that they received was requested. Source of fluoride ingestion from drinking water was assessed by asking *“Where does your family get the drinking water?”* The options were 1 = tap, 2 = well, 3 = borehole, 4 = spring, 5 = lake, 6 = river, 7 = rain water, 8 = stream. During analysis, the categories were dichotomized into 0 = tap water (with option 1) and 1 = non tap water (with options 2, 3, 4, 5, 6 and 7). In addition, we asked if their family uses magadi/masala/ginger as food additives when cooking. The options were “yes” or “no” for each item. During analysis the items were dichotomized into 0 = family do not use magadi and 1 = family uses magadi. Use of teeth in daily activities was assessed by asking *“Do you use your teeth in any habit like: nail biting, pen/pencil biting, opening soda, holding needles, chewing sticks/roots/sunflower seeds etc.”?* The responses were *“yes”* or *“no”.* As described elsewhere [28], prevalence of temporomandibular disorder (TMD) was assessed by asking two validated epidemiological questions: “*Do you have pain in your temple, face, jaw or jaw joint once a week or more?”* and *“Does it hurt once a week or more when you open your mouth or chew?”* The response was either “*yes”* or *“no”* and by having a positive response to one or all of the two questions was considered affirmative to TMD diagnosis [29].

In the multiple variable logistic regression analyses, each outcome variable had two levels/categories. Oral hygiene status was dichotomized into 0 = poor oral hygiene and 1 = good oral hygiene; gingival bleeding was dichotomized into 0 = without bleeding and 1 = with bleeding; dental caries was dichotomized into DMFT = 0 and DMFT > 0 and dental fluorosis was dichotomized into 0 = TF score 0–4 and 1 = TF score 5–9. In addition, dental erosion was dichotomized into 0 = grade 0 and 1 = grade 1–4; tooth wear was dichotomized into 0 = grade 0 and 1 = grade 1–4; and TMD was dichotomized into 0 = without TMD symptoms and 1 = with TMD symptoms.

### Oral clinical examination

The principal investigator (LS) performed all clinical examinations. The participant was examined in natural day light under field conditions, sitting on a chair, outside or inside the classroom. The dentition was cleaned and dried by sterile gauze and isolated by cotton rolls, if necessary. Disposable mouth mirrors and Sickle probe (No. 23 explorer or Shepherd’s hook) were used. Full report over the clinical findings has been reported elsewhere [28].

The Simplified Oral Hygiene Index (OHI-S) was used to assess the oral hygiene status [30]. This method also has been described elsewhere [28]. The scores were (0) for no plaque/calculus present, (1) for plaque or supra-gingival calculus covering not more than one third of the tooth surface, (2) for plaque or supra-gingival calculus covering more than one third but less than two thirds of the tooth surface, and (3) for plaque or supra-gingival calculus covering more than two thirds of the tooth surface.

Gingival Bleeding Index (GBI) was used to assess the gingival health [31]. Dental caries was assessed according to criteria specified by WHO, 2013 [32] and dental fluorosis by Thylstrup- Fejerskov - index (TF-index) on all buccal surfaces, except wisdoms teeth [33]. Dental erosion was partially recorded according to Johansson et al. 1996 [34] for palatal and facial surfaces of maxillary anterior teeth, and by the scale of Hasselkvist et al. [35] for grading cuppings of first molars. Tooth wear was graded as a full mouth recording of occlusal/incisal surfaces of all teeth according to Carlsson et al. 1985 [36].

### Statistical analysis

The Statistical Package for Social Sciences (SPSS) for PC version 25 (IBM corporation, Armonk, NY, USA) and STATA 15.0 (Stata corporation, Lakeway drive college station, Texas, USA) were used for data analysis. Descriptive statistics were carried out, followed by bivariate analysis using cross tabulations and Pearson’s chi-square statistical test. Intra-examiner concordances were examined using percentage agreement and Cohen’s Kappa. Multiple variable logistic regression analyses were performed to assess associations between oral health behaviors and oral diseases/conditions whilst adjusting for potential confounding factors in terms of socio-demographics (sex, age group, district, ethnicity, wealth index and maternal education) and using odds ratio (OR) and 95% confidence intervals (CI). Oral health related behaviors, significantly associated with oral diseases/conditions in unadjusted bivariate analyses were included in the multiple logistic regression analyses. The analyses were adjusted for the cluster of school, the primary sampling unit in this study. Level for statistical significance was set to *p* < 0.05.

## Results

### Sample characteristics

A total of 906 adolescents attending primary school grade 6 participated in this study (response rate 91.6%). Initially, a total of 989 adolescents were invited to participate, out of which 59 (6.0%) were absent during interviewing and 24 (2.6%) were excluded during analysis because of too high or low age. The age range finally accepted for participation in the study was 12–17 years (mean age 13.4 years, SD 1.2) and 56.1% of those were females. A total of 52.9 and 47.1% of the adolescents were from Monduli and Longido districts, respectively. Among the participants, 79.6% were from the Maasai ethnic group and 20.4% from the non Maasai ethnic group. For details of sociodemographic features and oral diseases/conditions frequency distribution in the total sample see Table 1.

**Table 1:** Frequency distribution of sociodemographic features and oral diseases/conditions in a total sample, *N* = 906

Sociodemographic feature	Category	Frequency % (*n*)
District of residence	Monduli	52.9 (479)
	Longido	47.1 (427)
Sex	Male	43.9 (398)
	Female	56.1 (508)
Age	12–14 years	87.3 (777)
	15–17 years	12.7 (113)
Ethnicity	Maasai	79.6 (721)
	Non Maasai	20.4 (185)
Wealth Index	Most poor	48.8 (438)
	Least poor	51.2 (459)
Mother’s education	Low (≤ primary school)	95.0 (861)
	High (≥ secondary school)	5.0 (45)
Oral hygiene status	Poor (OHI-S ≥ 1)	65.6 (594)
	Good (OHI-S < 1)	34.4 (312)
Gingival bleeding	Negative	59.1 (535)
	Positive	40.9 (371)
Dental caries	DMFT = 0	91.2 (826)
	DMFT > 0	8.8 (80)
Dental fluorosis	TF score 0	10.3 (93)
	TF score 1–9	89.7 (813)
Dental fluorosis	TF score 0–4	51.4 (466)
	TF score 5–9	48.6 (440)
Dental erosion	Grade 0–2	98.1 (889)
	Grade 3–4 (extending into dentine)	1.9 (17)
Dental erosion	Grade 0	70.0 (634)
	Grade 1–4	30.0 (272)
Tooth wear	Grade 0–1	83.5 (757)
	Grade 2–4 (extending into dentine)	16.5 (149)
TMD pain	No	88.2 (799)
	Yes	11.8 (107)

### Reliability testing

During training for scoring of dental erosion, the inter-examiner (between LS and AKJ) Cohen’s Kappa for all examined teeth during examination was 0.82. Oral clinical examination was carried out by the principal investigator (LS). Duplicate clinical examinations were carried out with 93 randomly selected participants 3 weeks apart. Intra-examiner reliability as per Cohen’s Kappa value was 0.98 for DMFT, 0.87 for TF index and 0.69 for dental erosion.

### Oral health related behaviors by ethnic group

As shown in Tables 2 and 3, a majority of the oral health related behaviors investigated varied significantly (*p* < 0.001) with ethnic group belongingness with the exception of eating sweets, drinking sour milk, meat consumption, use of teeth for nail biting, pencil biting, opening soda and holding needle, eating vegetables and fruits, and having a behavior of clenching and/or grinding teeth. Totals of 32.6% of the Maasai and 47.0% of the non Maasai adolescents reported high frequency of intake of biscuits. The ethnic distribution for use of plastic toothbrush and chewing sticks were respectively, 39.8% versus 74.6% (*p* < 0.001) and 53.7% versus 21.1% (*p* < 0.001) for Masaai and non-Masaai, respectively.

**Table 2:** Frequency distribution of oral health related behaviors (dietary and oral hygiene) overall and by ethnic groups

Variable	Overall (*N* = 906) % (*n*)	Maasai (*n* = 721) % (*n*)	non Maasai (*n* = 185) % (*n*)	*p*-value*
Frequency of: Eating sweets				
Low (≤1per week)	60.8 (551)	62.4 (450)	54.6 (101)	
High (≥2 per week or daily)	39.2 (355)	37.6 (271)	45.4 (84)	0.052
Eating biscuits				
Low (≤1per week)	64.5 (584)	67.4 (486)	53.0 (98)	
High (≥2 per week or daily)	35.5 (322)	32.6 (235)	47.0 (87)	< 0.001
Drinking carbonated soft drinks				
Low (≤1per week)	66.3 (601)	70.2 (506)	51.4 (95)	
High (≥2 per week or daily)	33.7 (305)	29.8 (215)	48.6 (90)	< 0.001
Drinking fruit drink				
Low (≤1per week)	68.7 (622)	71.8 (518)	56.2 (104)	
High (≥2 per week or daily)	31.3 (284)	28.2 (203)	43.8 (81)	< 0.001
Drinking tea with sugar				
Low (≤1per week)	21.7 (197)	24.7 (178)	10.3 (19)	
High (≥2 per week or daily)	78.3 (709)	75.3 (543)	89.7 (166)	< 0.001
Drinking tea without sugar				
Low (≤1per week)	88.7 (804)	87.5 (631)	93.5 (173)	
High (≥2 per week or daily)	11.3 (102)	12.5 (90)	6.5 (12)	0.021
Drinking milk from cows				
Low (≤1per week)	17.9 (162)	14.1 (102)	32.4 (60)	
High (≥2 per week or daily)	82.1 (744)	85.9 (619)	67.6 (125)	< 0.001
Drinking soured milk				
Low (≤1per week)	41.7 (378)	41.5 (299)	42.7 (79)	
High (≥2 per week or daily)	58.3 (528)	58.5 (422)	57.3 (106)	0.762
Eating meat				
Low (≤1per week)	55.4 (502)	56.7 (409)	50.3 (93)	
High (≥2 per week or daily)	44.6 (404)	43.3 (312)	49.7 (92)	0.115
Cleaning teeth				
Low (≤1per week)	24.6 (223)	27.2 (196)	14.6 (27)	
High (≥2 per week or daily)	75.4 (683)	72.8 (525)	85.4 (158)	< 0.001
Using toothpaste				
Low (≤1per week)	62.4 (565)	69.6 (502)	34.1 (63)	
High (≥2 per week or daily)	37.6 (341)	30.4 (219)	65.9 (122)	< 0.001
Type of tooth brush				
Plastic	46.9 (425)	39.8 (287)	74.6 (138)	
Chewing stick	47.0 (426)	53.7 (387)	21.1 (39)	
Charcoal	0.7 (6)	0.8 (6)	0.0 (0)	
Don’t have	5.4 (49)	5.7 (41)	4.3 (8)	< 0.001

*Pearson’s Chi-square teik

**Table 3:** Frequency distribution of oral health related behaviors (dental fluorosis, daily use of teeth as tool and TMD) overall and by ethnic groups

Variable	Overall (*N* = 906) % (n)	Maasai (*n* = 721) %(*n*)	Non Maasai (*n* = 185) %(*n*)	*p*-value*
Source of drinking water				
Tap water	56.7 (514)	49.8 (359)	83.8 (155)	
Non tap water	43.3 (392)	50.2 (362)	16.2 (30)	0.001
Family uses magadi				
No	59.9 (483)	57.7 (374)	69.0 (109)	
Yes	40.1 (323)	42.3 (274)	31.0 (49)	0.010
Do you use teeth for nail biting?				
No	54.9 (497)	55.9 (403)	50.8 (94)	
Yes	45.1 (409)	44.1 (318)	49.2 (91)	0.215
Do you use teeth for pen/pencil biting?				
No	58.1 (526)	58.9 (425)	54.6 (101)	
Yes	41.9 (380)	41.1 (296)	45.4 (81)	0.285
Do you use teeth for opening soda?				
No	48.2 (437)	48.1 (347)	48.6 (90)	
Yes	51.8 (469)	51.9 (374)	51.4 (95)	0.899
Do you use teeth for holding needle?				
No	57.9 (525)	57.6 (415)	59.5 (110)	
Yes	42.1 (381)	42.4 (306)	40.5 (75)	0.640
Do you use teeth for chewing sticks?				
No	57.1 (517)	55.2 (398)	64.3 (119)	
Yes	42.9 (389)	44.8 (323)	35.7 (66)	0.025
Do you use teeth for chewing roots?				
No	61.0 (553)	56.7 (409)	77.8 (144)	
Yes	39.0 (353)	43.3 (312)	22.2 (41)	< 0.001
Do you use teeth for chewing sunflower?				
No	57.3 (519)	58.9 (425)	50.8 (94)	
Yes	42.7 (387)	41.1 (296)	49.2 (91)	0.046
Do you eat vegetables?				
No	11.5 (104)	11.8 (85)	10.3 (19)	
Yes	88.5 (802)	88.2 (636)	89.7 (166)	0.563
Do you eat fruits				
No	4.6 (42)	5.0 (36)	3.2 (6)	
Yes	95.4 (864)	95.0 (685)	96.8 (179)	0.313
Do you clench or grind your teeth?				
No	64.8 (587)	64.8 (467)	64.9 (120)	
Yes	19.1 (173)	19.0 (137)	19.5 (36)	
I don’t know	16.1 (146)	16.2 (117)	15.7 (29)	0.978

*Pearson’s Chi-square test

### Oral diseases by oral health related behaviors

Socio-demographic and oral health related behaviors significantly associated with oral health outcomes in unadjusted analyses, were included as covariates in the multiple variable logistic regression. Tables 4, 5, 6 and 7 depicts unadjusted frequency distributions and the ORs and 95% CI for oral hygiene, gingival bleeding, caries experience, dental fluorosis, dental erosion, tooth wear and TMD by oral health related behaviors whilst adjusted for sociodemographic characteristics.

**Table 4:** Oral hygiene status and gingival bleeding according to oral health related behaviors. Unadjusted a Chi square and adjusted multiple variable logistic regression analyses

Dependent variable	Independent variables	Unadjusted analysis % (*n*)	Adjusted OR (95% CI)^a^
Oral hygiene status (poor hygiene)	Frequency of cleaning teeth		
	High (≥2 per week or daily)	56.4 (385)	1
	Low (≤1per week)	93.7 (209)*	10.0 (4.3–20.0)
	Type of cleaning instrument		
	Chewing stick	67.6 (288)	1
	Plastic	60.2 (256)*	0.9 (0.7–1.3)
Gingival bleeding (Yes)	Frequency of cleaning teeth		
	Low (≤1per week)	84.3 (188)	1
	High (≥2 per week or daily)	26.8 (183)*	0.1 (0.04–0.14)
	Type of cleaning instrument		
	Chewing stick	45.5 (194)	1
	Plastic	31.8 (135)*	0.7 (0.53–0.99)

*Significant Pearson Chi-square test (*p* < 0.05)

^a^Multiple variable logistic regression analyses were adjusted for sex, age and ethnicity

**Table 5:** Dental caries experience and dental fluorosis according to oral health related behaviors. Unadjusted Chi square and adjusted multiple variable logistic regression analyses

Dependent variable	Independent variables	Unadjusted analysis % (*n*)	Adjusted OR (95% CI)^a^
Dental caries experience (DMFT> 0)	Frequency of eating biscuits		
	Low (≤1per week)	5.8 (34)	1
	High (≥2 per week or daily)	14.3 (46)*	2.5 (1.7–3.8)
	Frequency of drinking tea with sugar		
	Low (≤1per week)	9.6 (19)	
	High (≥2 per week or daily)	8.6 (61)	
	Frequency of drinking milk from cows		
	Low (≤1per week)	5.6 (9)	
	High (≥2 per week or daily)	9.5 (71)	
	Frequency of tooth cleaning		
	Low (≤1per week)	9.0 (20)	
	High (≥2 per week or daily)	8.8 (60)	
Dental fluorosis (TF grade 5–9)	Source of drinking water		
	Tap water	41.2 (212)	1
	Non tap water	58.2 (228)*	1.3 (0.6–2.8)
	Family uses magadi		
	No	24.4 (118)	1
	Yes	90.4 (292)*	24.2 (11.6–50.6)

*Significant Pearson Chi-square test (*p* < 0.05)

^a^logistic regression analyses adjusted for district of residence, age and ethnicity

**Table 6:** Dental erosion and tooth wear according to oral health related behaviors. Unadjusted a Chi square and adjusted multiple variable logistic regression analyses

Dependent variable	Independent variables	Unadjusted analysis % (*n*)	Adjusted OR (95% CI)^a^
Dental erosion (grade 1–4)	Frequency of drinking carbonated soft drinks		
	Low(≤ once per week)	23.1 (139)	1
	High (≥ twice per week)	44.3 (135)*	1.6 (1.1–2.5)
	Frequency of drinking fruit drink		
	Low(≤ once per week)	24.0 (149)	1
	High (≥ twice per week)	44.0 (125)*	1.5 (1.0–2.2)
	Frequency of drinking milk from cows		
	Low (≤1per week)	35.8 (58)	1
	High (≥2 per week or daily)	29.0 (216)*	0.7 (0.5–1.0)
	Eating meat		
	Low (≤1per week)	25.3 (127)	1
	High (≥2 per week or daily)	36.4 (147)*	1.3 (0.9–2.0)
	Frequency of cleaning teeth		
	Low (≤1per week)	19.7 (44)	1
	High (≥2 per week or daily)	33.7 (230)*	1.7 (1.1–2.6)
	Frequency of using tooth paste		
	Low(≤ once per week)	25.5 (144)	1
	High (≥ 2x per week)	38.1 (130)*	1.1 (0.7–1.8)
Tooth wear (Grade 1–4)	Frequency of drinking carbonated soft drinks		
	Low(≤ once per week)	46.3 (278)	
	High (≥ twice per week)	45.6 (139)	
	Frequency of drinking fruit drink		
	Low(≤ once per week)	46.9 (292)	
	High (≥ twice per week)	44.0 (125)	
	Frequency of drinking milk from cows		
	Low (≤1per week)	50.0 (81)	
	High (≥2 per week or daily)	45.2 (336)	
	Frequency of cleaning teeth		
	Low (≤1per week)	51.1 (114)	1
	High (≥2 per week or daily)	44.4 (303)*	0.8 (0.6–1.1)
	Type of cleaning instrument		
	Chewing stick	50.9 (217)	1
	Plastic	40.7 (173)*	0.6 (0.4–0.8)
	Frequency of using tooth paste		
	Low(≤ once per week)	46.5 (263)	
	High (≥ 2x per week)	45.2 (154)	
	Uses teeth for nail biting		
	No	35.8 (178)	1
	Yes	58.4 (239)*	1.9 (1.4–2.4)
	Uses teeth for pen/pencil biting		
	No	41.4 (218)	1
	Yes	52.4 (199)*	0.9 (0.6–1.3)
	Uses teeth for opening soda		
	No	35.9 (157)*	1
	Yes	55.4 (260)	1.8 (1.4–2.4)
	Uses teeth for holding needles		
	No	38.1 (200)	1
	Yes	57.0 (217)*	1.6 (1.3–2.1)
	Uses teeth for chewing sunflower seeds		
	No	42.6 (221)*	1
	Yes	50.6 (196)	1.0 (0.8–1.3)

*Significant Pearson Chi-square test (*p* < 0.05)

^a^ Multiple variable logistic regression analyses were adjusted for ethnicity, maternal education and wealth index

**Table 7:** TMD pain according to oral health related behaviors. Unadjusted a Chi square and adjusted multiple variable logistic regression analyses

Dependent variable	Independent variables	Unadjusted analysis % (n)	Adjusted OR (95% CI)^a^
TMD (2Q/TMD > 0)^b^	Eating vegetables		
	No	13.5 (14)	
	Yes	11.6 (93)	
	Eating fruits		
	No	9.5 (4)	
	Yes	11.9 (103)	
	Do you clench or grind your teeth?		
	No	9.0 (53)	1
	Yes	23.1 (40)*	2.3 (1.5–3.7)

*Significant Pearson Chi-square test (*p* < 0.05)

^a^ Multiple variable logistic regression analyses were adjusted for district of residence, ethnicity and mother’s education

^b^ Two epidemiological questions regarding TMD

As shown in Table 4, frequency of tooth cleaning was statistically significantly associated with oral hygiene status, whereas frequency of tooth cleaning and type of toothbrush were significantly associated with gingival bleeding both in bivariate and multiple variable logistic regression analyses. As compared to adolescents reporting high frequency of tooth cleaning, those who reported low frequency were significantly more likely to present with poor oral hygiene status. The corresponding OR was 10.0 (95% CI 4.3–20.0). Adjusted ORs for presenting with gingival bleeding were 0.1 (95% CI 0.04–0.14) if reporting high versus low frequency of tooth cleaning and 0.7 (95% CI 0.53–0.99) if reporting use of plastic toothbrush versus chewing stick.

Frequency of eating biscuits was the only behavior that was significantly associated with caries experience in both unadjusted and adjusted analyses. As compared to adolescents reporting low frequency of intake, their counterparts who reported high intake frequency were 2.5 times (OR 2.5, 95% CI 1.7–3.8) more likely to present with DMFT > 0 after having adjusted for socio-demographic characteristics. Those who reported use of magadi, compared to those who did not, were more likely to present with dental fluorosis (TF 5–9, OR 24.2 (95% CI 11.6–50.6). For more details see Table 5.

In the adjusted regression analyses (Table 6), those who reported high frequency of intake of carbonated soft drinks (OR = 1.6, 95% CI 1.1–2.5) and cleaning teeth (OR = 1.7, 95% CI 1.2–2.6), were more likely to present with dental erosion. Biting nails (OR = 1.9, 95% CI 1.4–2.4), using teeth for opening soda and holding needles (OR = 1.8, 95% CI 1.4–2.4 and OR = 1.6, 95% CI 1.3–2.1, respectively), and using chewing stick type (OR = 1.7, 95% CI 1.3–2.5) were significantly associated to increased severity of tooth wear.

Adolescents who reported clenching and/or grinding teeth (OR = 2.3, 95% CI 1.5–3.7) were more likely to present with TMD pain compared to those who did not (Table 7).

## Discussion

This study aimed to explore the association between oral health related behavior and oral diseases in adolescents living in Maasai population areas of Tanzania. As earlier reported, poor oral hygiene, gingival bleeding and dental fluorosis were common findings among this group of adolescents while dental caries, dental erosion, tooth wear and TMD symptoms were less common. Ethnic disparities predominated with respect to gingival bleeding, dental caries and dental erosion [28]. In this study, there was a significant difference in some oral health related behavior between the Maasai and non Maasai groups. Low frequency of oral hygiene practices was associated with poor oral hygiene. High frequency of cleaning teeth and the use of plastic type of tooth brush were associated with less gingival bleeding. High consumption of biscuits was associated with presence of dental caries and the use of magadi as a food additive was associated with severe dental fluorosis (TF grade 5–9). Regular intake of carbonated soft drinks and regular tooth cleaning were covariates for dental erosion. Using teeth as a tool as for: biting nails, opening soda and holding needles were independently associated with tooth wear. Adolescents who reported to clench/grind their teeth was the only covariate for TMD.

Consistent with previous studies, low frequency of oral hygiene practices associated with poor oral hygiene and a high frequency of oral hygiene practices associated with less gingival bleeding [37, 38]. In addition a high frequency of intake of biscuits and carbonated soft drinks was associated with dental caries (DMFT> 0) and dental erosion, respectively [35, 39]. Not surprisingly, those who reported to clean their teeth less often, had poorer oral hygiene status and more gingival bleeding compared to those who cleaned more often [40]. But, although > 75% of the adolescents reported a high frequency of tooth cleaning, the majority of them (66%) had poor oral hygiene status. This imply that cleaning teeth twice to daily a week is not enough and/or that an ineffective cleaning technique was utilized or maybe there was over reporting of the frequency of oral hygiene habits.

In this study, the use of a wooden chewing stick (47%) was less common than previous reports from rural areas of Kenya (59%, 5–17 year olds) and Burkina Faso (64%, 12 year olds), but more common than reports from the rural areas of Tanzania (4–36%,5–22 year olds) [41–44]. On the other side, the use of a plastic tooth brush in this study (47%) was more common than reports from Kenya (41%) and Burkina Faso (36%) [43, 44], but less common than earlier reports from Tanzania (64–96%) [41, 42]. In this study, the use of a plastic tooth brush was correlated with less gingival bleeding. This finding is contrary to an earlier report from India where it was shown that there were no significant difference between the use of a chewing stick or a plastic toothbrush on gingival health [45]. Regarding ethnicity there was a significant difference between the ethnic groups in this study in terms of oral hygiene behavior. Compared to non Maasai group, the Maasai group reported to clean their teeth more irregularly and reported to use chewing sticks for cleaning teeth more regularly. The more common use of chewing sticks than plastic toothbrushes among the Masaai in this study might be a result of availability and economic considerations. In this regard, our previous report showed that the Maasai adolescents in this study were from more poor families compared to the non Maasais in terms of wealth index [28].

The low prevalence of dental caries found in this study (8.8%) [28] imply a low exposure to cariogenic diet, which is supported by the findings in this study where the majority (60.8–64.5%) of adolescents reported low intake of sweets and biscuits. The traditional diet for the Maasais is consisting of dairy products such as milk but also meat and blood from cattles, products with low cariogenic potential [46]. In this study, 82.1% of the adolescents reported high frequency of drinking fresh milk and this might have contributed to the low prevalence of dental caries among the adolescents [47]. In addition, a high fluoride content in their drinking water and using Magadi as food additive [48] may also have reduced their risk for dental caries [49]. Considering ethnicity, more adolescents from Maasai group (85.9%) reported regular intake of fresh milk from cows and regular intake of magadi as food additives compared to non Maasais. Probably due to this, it is therefore not surprising to find that more Maasais were less affected by dental caries compared to non Maasais [28].

In this study a correlation between severe dental fluorosis and the use of Magadi as food additive was found, similar to other studies [11, 50]. It is known that Magadi from Tanzania contain a high concentration of fluoride (160 to 1750 mg/l) [51]. The Maasai group reported the use of Magadi more often than the non Maasais which may be one explanation for their higher prevalence and severity of dental fluorosis [28].

Probably the rural environment and poor economic situation in which study participants live might limit their access to erosive conducive challenges. But even so, about one third of all study participants reported to drink carbonated soft drinks and/or fruit juices regularly. Our previous study [28] revealed low prevalence (1.9%) of dental erosion extending into dentine and there was a significant association between high frequency intake of carbonated soft drinks and dental erosion in this study, similar to others [35, 52]. This study revealed also, in agreement with previous reports, an association between dental erosion and oral hygiene [53, 54]. In this regard, the Maasai adolescents reported significantly lesser teeth cleaning and use of toothpaste than non Maasais. Subsequently, the difference in dietary habits between the two ethnic groups in addition to more irregular oral hygiene practices may also explain why Maasais were less affected by dental erosion compared to non Maasais [28].

The severity and pattern of tooth wear could be influenced not only by acidic challenges such as soft drinks but also factors like abrasiveness of the diet, parafunctional habits and/or the use of teeth as tools [55, 56]. Foods consumed in this society might often be hard and abrasive in nature, especially meat, since it is often mixed with ashes in the process of cooking and eaten when half cooked making it more abrasive to chew. The relative frequent intake of acidic drinks among the participants may also contribute to a worsening of the abrasive effect, resulting in more severe tooth wear [35]. About 45% of the study participants reported to use teeth for biting nails and 52% reported to use teeth for opening soda bottles. The excessive biting force required for exercising these habits and the abrasiveness of the material involved in the practice could also have contributed to relatively high prevalence of tooth wear. However, the tooth wear process has a multifactorial etiology and other not investigated factors may have played a role as well [55].

This study found that self-reported bruxism was associated with TMD similar with previous reports [57, 58]. Although previous reports have revealed that self-reported bruxism is associated with TMD, reports based on polysomnography (PSG) studies on sleep bruxism have not [59, 60]. In this study, other parafunctional habits apart from bruxism, were not associated with development of TMD similar to previous reports [61].

A clear majority (about 95%) of the adolescents in this study had never visited a dentist/dental therapist for toothache or checkup. The low dental service utilization indicates a need of actions that could facilitate access to these services. Oral health education is an essential and basic part of dental health care services as it provides information empowering people to raise awareness leading to the adoption of healthier lifestyles, positive attitudes and good oral health behaviors. It has been reported that oral health education is a powerful and successful tool in promoting oral health in adolescents [62] and that school provides a good setting for health-education programs [63]. In sub Saharan Africa, the health technology assessment (HTA) reports is scarce due to lack of capacity to undertake HTA and lack of high-quality data and lack of research evidence [64]. However, HTA reports from WHO suggests that structured research methodology and transparent and health procedures, including preventive intervention, helps to obtain information for evaluation of safety, cost-effectiveness, quality, and efficiency dimensions of such techniques when used in the health systems [65].

The strengths with the present study are the random sampling of the primary sampling units (schools), which may permit the generalization of the results to the communities living in Maasai populated areas. Using the research assistants fluent in both Swahili and Maasai language to interview the adolescents ensured that the interviewee had understood the question. In addition, one examiner performed the clinical examinations of all children thus reducing any inter-examiner variability. On the other side, using one examiner only may lead to introduction of observer effect bias where the observer may influence the participants of the study. Also it can lead to confirmation bias whereby the experinmenter interprets the results incorrectly because of the tendency to look for information that conforms to their hypothesis, and overlook information that argues against it [66]. However, since most of the background factors, such as dietary habits were based on self-report, it is always a possibility that the child might have misunderstood the question, forgotten or that the response might be influenced by social desirability. The cross-sectional method was utilized in data collection, making difficulties in establishing a causal relationship.

## Conclusion

Oral health related behaviors have a significant impact on oral diseases/conditions among adolescents attending primary schools in Maasai population areas with obvious differences in behavior between the Maasai and non Maasai ethnic groups. There is a need for addressing oral health and to encourage behaviors that promote good oral health and dental care service utilization in this society.

## Data Availability

The datasets used and/or analyzed during the current study are available from the corresponding author on request.

## Abbreviations

CI: Confidence Interval
DMFT: Decayed Missing Filled Tooth
GBI: Gingival Bleeding Index
HTA: Health Technology Assessment
OHI-S: Simplified Oral Hygiene Index
OR: Odds Ratio
SD: Standard Deviation
SPSS: Statistical Package for Social Sciences
TF-index: Thylstrup-Fejerskov-index
TMD: Temporomandibular Disorder
WHO: World Health Organisation
